# Resveratrol modulates the Akt/GSK-3β signaling pathway in a middle cerebral artery occlusion animal model

**DOI:** 10.1186/s42826-019-0019-8

**Published:** 2019-10-15

**Authors:** Dong-Ju Park, Ju-Bin Kang, Fawad-Ali Shah, Phil-Ok Koh

**Affiliations:** Department of Anatomy, College of Veterinary Medicine, Research Institute of Life Science, Gyeongsang National University, 501 Jinju-daero, Jinju, 52828 South Korea

**Keywords:** Akt, GSK-3β, MCAO, Resveratrol

## Abstract

Cerebral ischemia is a major cause of neurodegenerative disease. It induces neuronal vulnerability and susceptibility, and leads to neuronal cell death. Resveratrol is a polyphenolic compound that acts as an anti-oxidant. It exerts a neuroprotective effect against focal cerebral ischemic injury. Akt signaling pathway is accepted as a representative cell survival pathway, including proliferation, growth, and glycogen synthesis. This study investigated whether resveratrol regulates Akt/glycogen synthase kinase-3β (GSK-3β) pathway in a middle cerebral artery occlusion (MCAO)-induced ischemic brain injury. Adult male rats were intraperitoneally injected with vehicle or resveratrol (30 mg/kg) and cerebral cortices were isolated 24 h after MCAO. Neurological behavior test, corner test, brain edema measurment, and 2,3,5-triphenyltetrazolium chloride staining were performed to elucidate the neuroprotective effects of resveratrol. Phospho-Akt and phospho-GSK-3β expression levels were measured using Western blot analysis. MCAO injury led to severe neurobehavioral deficit, infraction, and histopathological changes in cerebral cortex. However, resveratrol treatment alleviated these changes caused by MCAO injury. Moreover, MCAO injury induced decreases in phospho-Akt and phospho-GSK-3β protein levels, whereas resveratrol attenuated these decreases. Phosphorylations of Akt and GSK-3β act as a critical role for the suppression of apoptotic cell death. Thus, our finding suggests that resveratrol attenuates neuronal cell death in MCAO-induced cerebral ischemia and Akt/GSK-3β signaling pathway contributes to the neuroprotective effect of resveratrol.

## Introduction

Resveratrol is a polyphenolic compound that abundantly exists in grape and red wine. It is well known that resveratrol acts as an anti-oxidant and prevents oxidative damage in vascular and nervous system [1–3]. It also has a vasodilatory effect through stimulating nitric oxide production in endothelial cells [4]. Moreover, it exerts a neuroprotective effect against traumatic brain injury. Neuroprotective mechanisms of resveratrol are associated with its anti-inflammatory, anti-apoptotic, and anti-oxidant properties [5–7]. Resveratrol also plays a critical role in alleviation of neurodegenerative diseases such as Parkinson disease and Alzheimer’s disease [8, 9]. Resveratrol protects neurons against focal cerebral ischemia and attenuates brain damage via activation of PI3K/Akt signaling pathway [10, 11].

Akt is a member of serine/threonine protein kinases family that performs an essential role in the modulation of cell development, proliferation, growth, and survival [12]. Akt is known as protein kinase B and extensively expressed in various tissues. Akt acts as a mediator of cell survival and regulates numerous downstream targets, such as glycogen synthase kinase-3 (GSK-3), forkhead transcription factors, and Bad [13–15]. It is also involved in multiple cellular processes, such as cell proliferation, transcription, apoptosis, and glucose metabolism [15, 16]. Moreover, Akt has a critical function in neuronal survival during apoptotic injury [17]. Akt is substantially activated in the nervous system during cellular stress. Overexpression of Akt prevents apoptosis caused by growth factor depletion in cerebellar granule neurons [18].

GSK-3 is a serine/threonine protein kinases that regulates glycogen synthesis in response to insulin [19]. Moreover, it phosphorylates a broad range of substrates, including transcription factors, translation initiation factor, and eukaryotic initiation factor 2 [20, 21]. GSK-3 has two isoform, GSK-3α and GSK-3β. GSK-3β is a critical downstream target of the phosphoinositide 3-kinase (PI3K)/Akt signaling pathway and activity of GSK-3β is suppressed by Akt-mediated phosphorylation at serine 9 [19, 22]. GSK-3β activates caspase-3 and leads to apoptotic cell death. Activated Akt inhibits GSK-3β function and mediates anti-apoptotic effect [23]. Previous studies reported that resveratrol improves neurological behavior score and attenuates brain damage on ischemic stroke [24, 25]. Although many studies have been shown the neuroprotective effects of resveratrol, its mechanism has not been completely elucidated. Thus, this study confirmed the neuroprotective effects of resveratrol in middle cerebral artery occlusion (MCAO) animal model and investigated the underlying mechanisms of resveratrol through phosphorylations of Akt and its downstream target, GSK-3β.

## Materials and methods

### Experimental animals

Adult male Sprague-Dawley rats (220–230 g, *n* = 32) were purchased from Samtako Co. (Animal Breeding Center, Osan Korea). All animals were housed in controlled light condition (12 h light/ 12 h dark cycle) and temperature (25 °C). Drinking water and feed were freely supplied**.** All experimental procedures were carried out according to the guideline of the Institutional Animal Care and Use Committee of Gyeongsang National University. Animals were divided into the following four groups: vehicle + sham, resveratrol + sham, vehicle + MCAO, and resveratrol + MCAO (8 rats per group). Resveratrol (30 mg/kg, Sigma-Aldrich, St. Louis, MO, USA) was dissolved in 0.05% dimethyl sulfoxide (DMSO; Sigma-Aldrich) in phosphate buffered saline and immediately intraperitoneally injected [26]. Vehicle-treated animals were injected with only DMSO solution without resveratrol.

### Middle cerebral artery occlusion

Rats were anesthetized with Zoletil (50 mg/kg; Virbac, Carros, France) and placed on a surgical plate with supine position. MCAO was surgically operated as previously described method [27]. A midline of neck skin was incised and right common carotid artery (CCA) was exposed. Right CCA was carefully separated from the adjacent muscles and nerves, and temporally blocked with a microvascular clip. Branches of external carotid artery (ECA) were carefully dissected and ligated. Proximal end of the ECA was incised and a nylon filament 4/0 with flame-rounded tip was slowly inserted into the incision of ECA. Nylon filament was advanced into the lumen of internal carotid artery (ICA) until a slight resistance felt. It was inserted approximately 21 to 23 mm from the bifurcation of ECA and occluded the origin of middle cerebral artery. Filament was ligated with ECA and skin incision was sutured with black silk. Sham-operated animals were performed with the same surgical procedure without insertion of a nylon filament. Animals were kept on a heating pad to maintain body temperatures and given free access to feed and water. Neurological behavior test was performed 24 h after MCAO and expeditiously **decapitated to reduce suffering.** Whole brains were carefully removed and cerebral cortices were separated. Tissues were kept at − 70 °C or fixed in 4% neutral buffered paraformaldehyde (NBF) for further experimental procedure.

### Neurological behavior test and brain edema measurement

Neurological behavior test was performed by previously described methods [28, 29]. This test was scored with a five-point scoring system by following standard: no neurological deficit (0), normal posture but failed to extend forepaw on the contralateral side of ischemic region (1), normal posture and circling to the contralateral side of ischemic region (2), falling down to the contralateral side of ischemic region (3), no impulsive movement (4). Isolated right cerebral cortices were weighed for evaluation of brain edema. Measured weight was considered as wet weight. After weight measurement, right cerebral cortices were dried for 24 h at 100 °C and weighed. This weight was taken as dry weight. The water content in the cerebral cortex was calculated as follows: [(wet weight - dry weight)/wet weight] × 100.

### Corner test

Corner test was performed to evaluate sensorimotor function by previously described manuals [30, 31]. Corner test apparatus was formed with two vertical boards (30 × 20 × 1 cm) that attached to each edge at the angle of 30° with a small opening. The gap between two boards encouraged animals to move toward to the corner. When animals move to the corner, both side of vibrissae simultaneously touched boards. After vibrissae stimulation, animals rear against the corner and turn right or left side. Turning movement is recorded and rearing movement is excluded from measurement. Ten turns were counted for each trial and result was expressed as the number of right turn [30, 31].

### Triphenyltetrazolium chloride staining

Whole brains were carefully removed from skull and sliced into 2 mm coronary sections with a brain matrix (Ted Pella, Redding, CA, USA). Coronary section levels were marked using bregma level. Sliced tissues were reacted in 2% triphenyltetrazolium chloride (TTC; Sigma Aldrich) for 30 min at 37 °C and fixed in 4% NBP for 24 h. Images of stained tissues were scanned with Agfa ARCUS 1200™ (Agfa-Gevaert, Mortsel, Belgium) and analyzed using Image-ProPlus 4.0 software (Media Cybernetics, Silver Spring, MD, USA) to evaluate the infarct volume. Ischemic area (%) was calculated by the following formula: (infarction area/whole section area) × 100.

### Hematoxylin and eosin staining

Fixed brain tissues were washed with flowing tap water, dehydrated by series of graded ethyl alcohol from 70 to 100%, and cleaned with xylene. Brain tissues were embedded in paraffin tank using embedding center (Leica, Westlar, Germany) and hardened as tissue blocks. Tissues were cut into 4 μm sections and placed on glass slides in tissue bath (Leica). Sections were dried on slide warmer (Thermo Fisher Scientific, Waltham, MA, USA), deparaffinized with xylene, and rehydrated by series of graded ethyl alcohol from 100 to 70%. They were stained with hematoxylin solution (Sigma-Aldrich) and eosin solution (Sigma-Aldrich), subsequently. Stained tissues were washed with tap water and dehydrated with series of graded ethyl alcohol. They were mounted with permount mounting solution (Thermo Fisher Scientific) and photographed using Olympus microscope (Olympus, Tokyo, Japan).

### Western blot analysis

Right cerebral cortices were homogenized in lysis buffer [1% Triton X-100, 1 mM EDTA in PBS (pH 7.4)] containing 0.2 mM phenylmethylsulfonyl fluoride. Homogenates were sonicated and centrifuged at 15,000 g for 30 min at 4 °C. Supernatants were collected and protein concentrations were determined with a bicinchoninic acid protein assay kit (Pierce, Rockford, IL, USA) according to the **manufacturer’s manual.** Protein samples were denatured by heating for 3 min at 100 °C and cooled for 1 min in ice. Equal amount of proteins (30 μg) were loaded and electrophoresed in 10% **sodium** dodecyl sulfate polyacrylamide gel electrophoresis gel. Separated proteins were transferred to polyvinylidene fluoride membranes. Membranes were incubated in 5% skim milk with Tris-buffered saline containing 0.1% Tween-20 (TBST) for 1 h. After washing with TBST, membranes were incubated for overnight at 4 °C with following primary antibodies: anti-Akt, anti-phospho-Akt (Serine 473), anti-GSK-3β, anti-phospho-GSK-3β (Serine 9), (diluted 1:1000, Cell Signaling Technology, Beverly, MA, USA), and anti-β-actin (diluted 1:1000, Millipore, Billerica, MA, USA). Membranes were washed three times with TBST for 10 min and treated with their respective secondary antibody (horseradish peroxidase-conjugated anti-rabbit IgG or anti-mouse IgG, diluted 1:5000, Cell Signaling Technology) for 2 h at room temperature. After washing with TBST, membranes were reacted with enhanced chemiluminescence Western Blotting detection reagents (GE Healthcare, Chicago, IL, USA) for the detection of immunoreactive protein bands. Membranes were exposed on Fuji medical X-ray film (Fuji Film, Tokyo, Japan) to visualize immunoreactive bands. The intensity value of protein bands were analyzed with Image J software (National Institutes of Health, Bethesda, MD, USA).

### Statistical analysis

**All experiment data were presented as the mean ± standard error of means (S.E.M.).** The results of each group were compared by two-way analysis of variance (ANOVA) followed by *post-hoc* Scheffe’s test. *P* < 0.05 was regarded as statistically significant.

## Results

**Fig. 1:**
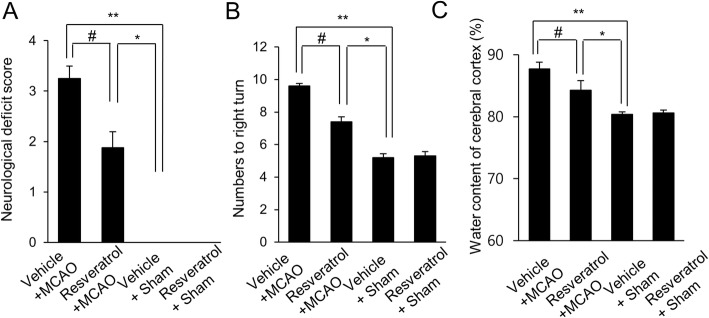
Neurobehavioral scores (**a**), corner test (**b**), and brain edema measurement (**c**) in vehicle + sham, resveratrol + sham, vehicle + middle cerebral artery occlusion (MCAO), and resveratrol + MCAO animals. Resveratrol attenuated the neurological behavior deficits and brain edema induced by ischemic stroke. Data (*n* = 8) are represented as the mean ± S.E.M. * *p* < 0.01, ** *p* < 0.05 vs. vehicle + sham animals, # *p* < 0.05 vs. vehicle + MCAO animals

We confirmed that MCAO-induced cerebral ischemia leads to neurological behavior dysfunction and brain infarction. As a further study, we showed that resveratrol prevented MCAO-induced these changes. We evaluated neurological damage by neurological behavior deficit scoring and brain edema measurement. MCAO-operated animals with vehicle showed severe neurological symptoms, such as involuntary circling and seizure. Resveratrol treatment in MCAO-operated animals attenuated these symptoms and showed only mild neurological symptoms. Resveratrol significantly reduced MCAO-induced increase in neurological deficit scores. Neurological deficit scores were 3.25 ± 0.24 and 1.87 ± 0.38 in vehicle+ MCAO and resveratrol + MCAO animals, respectively (Fig. 1a). Results of corner test showed the direction bias of the response by bilateral stimuli. The number of right turn indicates the ipsilateral side of insulted brain hemisphere. Sham-operated animals appeared as a similar pattern in turning left and right direction. However, MCAO animals with vehicle showed rightward-preferred turning pattern, resveratrol treatment reduced the number of rightward-preferred turning. Numbers of right turn were 9.45 ± 0.35 and 7.1 ± 0.53 in vehicle+ MCAO and resveratrol + MCAO animals, respectively (Fig. 1b). Water contents of cerebral cortices were measured to assess the degree of brain edema. MCAO-operated animals with vehicle showed severe brain edema, resveratrol treatment alleviated MCAO-induced excessive brain edema. Water contents were 87.73 ± 1.12 and 84.32 ± 1.51% in vehicle + MCAO and resveratrol + MCAO animals, respectively (Fig. 1c).

**Fig. 2:**
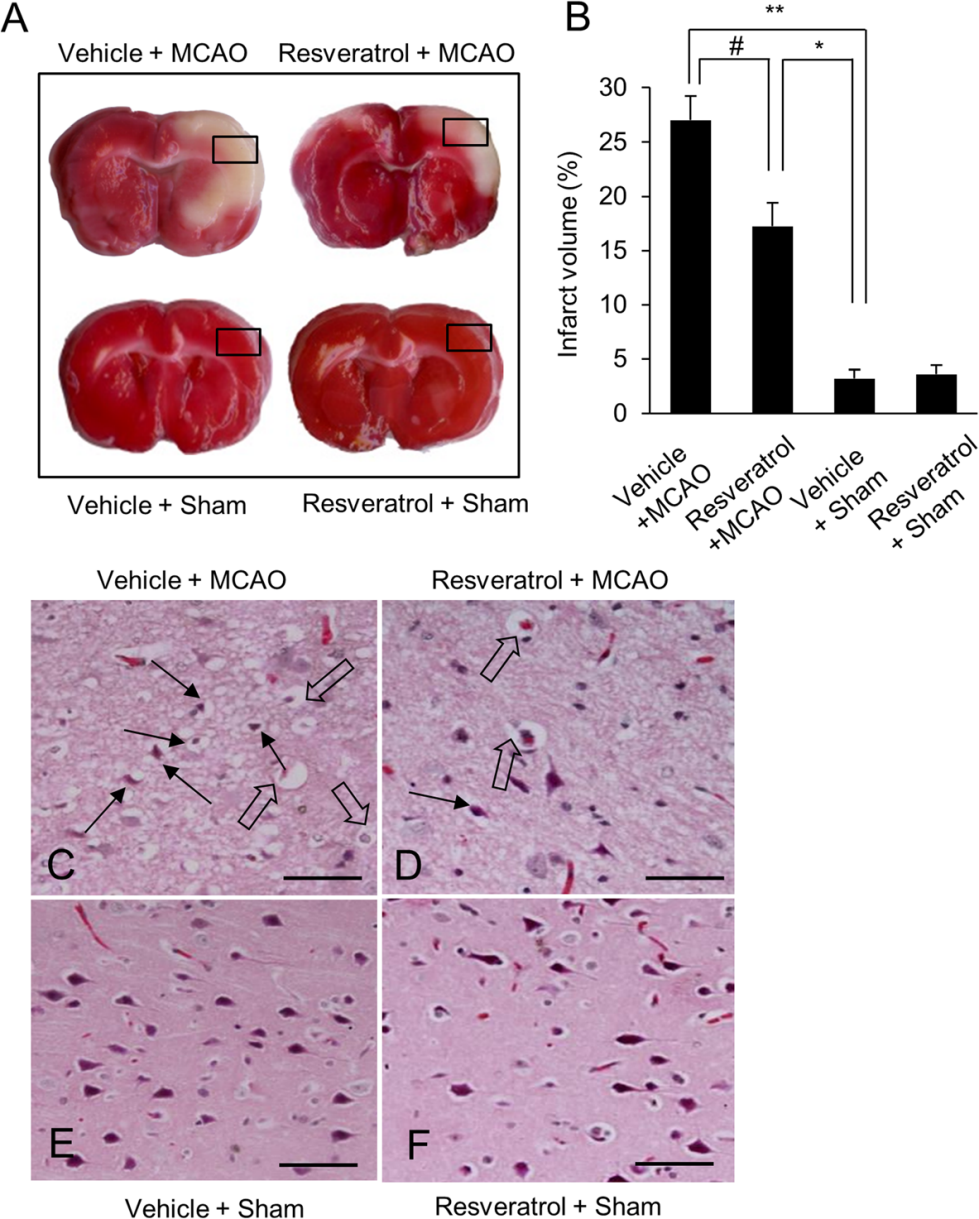
Representative photograph of TTC staining (**a**), infarct volume (**b**), and hematoxylin and eosin staining (**c**-**f**) in cerebral cortex of vehicle + sham, resveratrol + sham, vehicle + middle cerebral artery occlusion (MCAO), and resveratrol + MCAO animals. Infarct volume was calculated by ratio of infarction area to total area. Resveratrol attenuated the MCAO-induced infarct region. C-F photos indicate the square areas of A. Arrows indicate shrunken and condensed nuclei and open arrows indicate swelled and vacuolated forms. Scale bar = 100 μm. Data (*n* = 4) are represented as the mean ± S.E.M. * *p* < 0.01, ** *p* < 0.05 vs. vehicle + sham animals, # *p* < 0.05 vs. vehicle + MCAO animals

TTC staining was performed to evaluate the infarct volume. Infarct area was stained distinct red color, while infarct area remained as unstained white color. Results of TTC staining showed a widespread infarction in vehicle + MCAO animals. However, infarct area was significantly reduced in resveratrol + MCAO animals. Moreover, infarct area was not observed in sham-operated animals regardless of vehicle or resveratrol treatment (Fig. 2a). Volumes of infarct area were 26.31 ± 2.31% and 17.08 ± 2.70% in vehicle+ MCAO and resveratrol + MCAO animals, respectively (Fig. 2b). Morphological study showed the histopathological changes in cerebral cortex of MCAO-operated animals (Fig. 2c-f). We observed shape of typical pyramidal cells with large and round nucleus in sham-operated animals. They also had intact cytoplasm and dendrites. However, we found shrunken neurons with abnormal morphology in MCAO-operated animals with vehicle. They had condensed and shrunken nuclei and numerous vacuoles in cytoplasm. Resveratrol treatment attenuated MCAO-induced these pathological changes. Pyknotic nuclei and vacuoles in cytoplasm were reduced in MCAO animals with resveratrol.

**Fig. 3:**
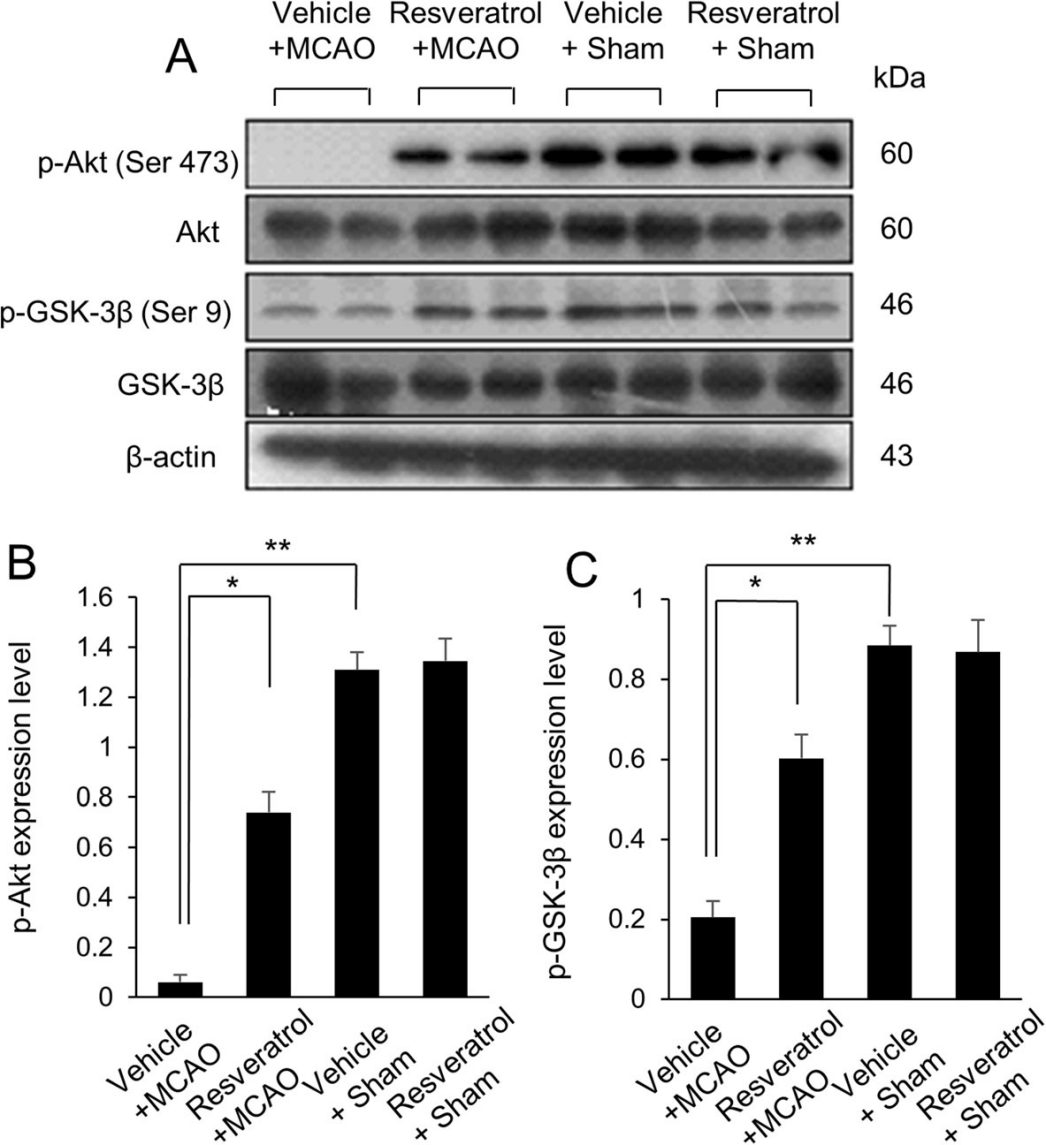
Western blot analysis of phospho-Akt and phospho-GSK-3β in cerebral cortex of vehicle + sham, resveratrol + sham, vehicle + middle cerebral artery occlusion (MCAO), and resveratrol + MCAO animals (**a**). Densitometric analysis is represented as a ratio of phospho-Akt (**b**) and phospho-GSK-3β (**c**) intensity to β-actin intensity. Data (*n* = 4) are shown as the mean ± S.E.M. * *p* < 0.01, ** *p* < 0.05 vs. vehicle + sham animals, # *p* < 0.05 vs. vehicle + MCAO animals

Western blot analysis result showed the expression changes of phospho-Akt and phospho-GSK-3β in cerebral cortex between vehicle + MCAO and resveratrol + MCAO animals (Fig. 3). phospho-Akt and phospho-GSK-3β protein expression levels were significantly decreased in cerebral cortex of vehicle + MCAO animals. However, these decreases were recovered in resveratrol + MCAO animals (Fig. 3a). Phospho-Akt levels were 0.06 ± 0.02 in vehicle + MCAO and 0.74 ± 0.08 in resveratrol + MCAO animals. Phospho-GSK-3β levels were 0.30 ± 0.02 and 0.61 ± 0.06 in vehicle + MCAO and resveratrol + MCAO animals, respectively (Fig. 3b).

## Discussion

Cerebral ischemia causes various neurological dysfunctions, including complex neuromuscular dysfunction and neurological behavior deficit [32]. It leads to the formation of pathological lesions, such as swollen axon, neuronal perikaryal damage, and brain edema [33, 34]. Moreover, it is accepted that free radical causes neuronal damage in cerebral ischemia. Resveratrol is a strong anti-oxidant that abundantly presents in grape seed. It suppresses infarct size and improves neurological functions in ischemic brain injury [24, 35–37]. In our study, we indirectly treated resveratrol through intraperitoneal injection. It is reported that resveratrol can distribute in brain through intraperitoneal injection [38]. Resveratrol also has a neuroprotective effect through intraperitoneal injection against kainic acid-induced brain damage, global cerebral ischemia and focal ischemia [38, 39]. Also, resveratrol exerts neuroprotective effect against cerebral ischemic injury by modulating mitochondrial dysfunction [37]. Resveratrol protects neurons against cerebral ischemia by inhibiting inflammation and apoptosis [40]. Moreover, it also prevents brain damage by reducing oxidative stress and ameliorating mitochondria damage in a cerebral ischemia [41]. We confirmed the neuroprotective effect of resveratrol in MCAO animal model. Resveratrol alleviated MCAO-induced neurological behavior deficits. Moreover, our previous study has been shown that resveratrol regulates the expression of various proteins that associated with oxidative stress in focal cerebral ischemia [26].

It is accepted that resveratrol exerts a neuroprotective effect by modulating of various molecular mechanism. Resveratrol decreases the elevated level of matrix metalloproteinase 9 caused by cerebral ischemia [42]. Moreover, resveratrol prevented the brain from ischemia through modulation of Ca^2+^ response element-binding protein [43]. Resveratrol protects hippocampal neurons against ischemic injury-induced damage via the extracellular signal regulated kinase signaling pathway [44]. It also exerts its neuroprotective effect by modulating of PI3K/Akt signaling pathway [11]. Resveratrol also attenuates ischemic brain damage through upregulation of inflammatory factors, interleukin-1 beta (IL-1β) and tumor necrosis factor-alpha (TNF-α) [11]. PI3K/Akt signaling pathway plays a key role for cell growth and cell survival. Akt also exerts anti-apoptotic effect by inhibiting the pro-apoptotic proteins, such as Bad and forkhead transcription factors [14]. Akt prevents injury-induced neuronal death and accelerates axonal regeneration [45]. It also contributes to neuronal migration and survival [46–49]. Our previous studies demonstrated that MCAO decreases Akt phosphorylation and consecutively reduces phosphorylation of its down-stream targets such as Bad, forkhead transcription factors, and GSK-3 beta [50–53]. Moreover, neuroprotective agents alleviate MCAO-induced decreases in phosphorylation of Akt and its down-stream targets [50–53]. MCAO clearly results in decreased levels of phospho-Akt and phospho-GSK-3β and neuroprotective agents attenuate the decreases in the levels of these proteins [50, 53]. The present study showed that focal cerebral ischemia significantly reduces phospho-Akt and phospho-GSK-3β expressions in cerebral cortex. Moreover, resveratrol delays the progression of 6-hydroxydopamine-induced motor dysfunction and apoptosis by activating PI3K/Akt cell survival pathway [54]. Phosphorylation of Akt is upregulated in early stage of ischemic stroke as a defensive reaction of neuronal cells whether this upregulation is terminated in late stage of ischemic stroke [55]. In our study, we performed MCAO for 24 h which is a late stage of ischemic stroke and phosphorylation of Akt is suppressed. However, resveratrol attenuated MCAO-induced decrease in phospho-Akt. Decrease of phospho-Akt induces apoptotic cell death and consequently inhibits cell survival. In addition, we elucidated the changes of GSK-3β by resveratrol treatment in MCAO-induced cerebral ischemia. Our results showed that resveratrol treatment in cerebral ischemia regulates Akt and its downstream target, GSK-3β. Focal cerebral ischemia caused by MCAO decreases phospho-GSK-3β levels and resveratrol alleviates decreases in phospho-GSK-3β. GSK-3β induces cell death by increasing caspase-3 activity in ischemic injury [56]. Thus, it is considered that phosphorylation of GSK-3β by Akt is a critical process for the inhibition of its pro-apoptotic activity. Previous study showed that resveratrol significantly downregulates cleaved caspase-3 and bax expressions, upregulates bcl-2 expression in stroke condition [57]. Moreover, resveratrol alleviates nerve injury in cerebral ischemia via up-regulation of hippocampal Bcl-2 [58]. Inactivation of caspase-3 leads to the suppression of apoptotic cell death. Resveratrol improves neurological function and neuronal damage, alleviates neuronal apoptosis in cerebral ischemia. We clearly demonstrated that resveratrol prevents the ischemic injury-induced decrease in phospho-GSK-3β expression. The maintenance of phospho-GSK-3β by resveratrol in cerebral ischemia mediates the inactivation of caspase-3 and suppression of apoptosis [56, 57]. The Akt/GSK-3β signaling pathway is an important mechanism of neuroprotective effect. We elucidated the fact that resveratrol prevents the MCAO injury-induced reductions in phospho-Akt and phospho-GSK-3β.

## Conclusions

In this study, we confirmed that resveratrol improves neuronal damage against MCAO-induced cerebral ischemic injury. Furthermore, our study reveals that resveratrol has a neuroprotective effects by regulating the Akt/GSK-3β signaling pathway. Therefore, this study can suggest the underlying mechanisms in neuroprotective effect of resveratrol attributing to neuronal cell survival.

## Data Availability

The data that support the findings of this study are available on request from the corresponding author on reasonable request.

## Abbreviations

CCA: Common carotid artery
DMSO: Dimethyl sulfoxide
ECA: External carotid artery
GSK-3: Glycogen synthase kinase-3
GSK-3β: Glycogen synthase kinase-3β
ICA: Internal carotid artery
IL-1β: Interleukin-1 beta
MCAO: Middle cerebral artery occlusion
NBF: Neutral buffered paraformaldehyde
NRF: National research foundation of korea
PI3K: Phosphoinositide 3-kinase
TBST: Tris-buffered saline containing 0.1% Tween-20
TNF-α: Tumor necrosis factor-alpha
TTC: Triphenyltetrazolium chloride
